# Recruitment Variability in North Atlantic Cod and Match-Mismatch Dynamics

**DOI:** 10.1371/journal.pone.0017456

**Published:** 2011-03-07

**Authors:** Trond Kristiansen, Kenneth F. Drinkwater, R. Gregory Lough, Svein Sundby

**Affiliations:** 1 Institute of Marine Research and Bjerknes Centre for Climate Research, Bergen, Norway; 2 Northeast Fisheries Science Center, National Marine Fisheries Service (NMFS), National Oceanic and Atmospheric Administration (NOAA), Woods Hole, Massachusetts, United States of America; National Oceanic and Atmospheric Administration/National Marine Fisheries Service/Southwest Fisheries Science Center, United States of America

## Abstract

**Background:**

Fisheries exploitation, habitat destruction, and climate are important drivers of variability in recruitment success. Understanding variability in recruitment can reveal mechanisms behind widespread decline in the abundance of key species in marine and terrestrial ecosystems. For fish populations, the match-mismatch theory hypothesizes that successful recruitment is a function of the timing and duration of larval fish abundance and prey availability. However, the underlying mechanisms of match-mismatch dynamics and the factors driving spatial differences between high and low recruitment remain poorly understood.

**Methodology/Principal Findings:**

We used empirical observations of larval fish abundance, a mechanistic individual-based model, and a reanalysis of ocean temperature data from 1960 to 2002 to estimate the survival of larval cod (*Gadus morhua*). From the model, we quantified how survival rates changed during the warmest and coldest years at four important cod spawning sites in the North Atlantic. The modeled difference in survival probability was not large for any given month between cold or warm years. However, the cumulative effect of higher growth rates and survival through the entire spawning season in warm years was substantial with 308%, 385%, 154%, and 175% increases in survival for Georges Bank, Iceland, North Sea, and Lofoten cod stocks, respectively. We also found that the importance of match-mismatch dynamics generally increased with latitude.

**Conclusions/Significance:**

Our analyses indicate that a key factor for enhancing survival is the duration of the overlap between larval and prey abundance and not the actual timing of the peak abundance. During warm years, the duration of the overlap between larval fish and their prey is prolonged due to an early onset of the spring bloom. This prolonged season enhances cumulative growth and survival, leading to a greater number of large individuals with enhanced potential for survival to recruitment.

## Introduction

The match-mismatch hypothesis is often used to explain annual variations in recruitment in both terrestrial and marine ecosystems [1]. However, there is evidence that a good match between prey and consumer does not necessarily lead to high recruitment [2]. Several additional physical (e.g. wind forcing, pollution) and biological (e.g. predators) factors can influence larval and juvenile mortality, thereby lowering recruitment potential [2], [3]. Nonetheless, a match in timing of larval and prey production has been shown to have the potential for positive effects on year-class strength [1]. However, a lack of observation and modeling data at large spatial scales has limited our understanding of the underlying mechanisms of match-mismatch dynamics. Using a mechanistic modeling approach our study focuses on how match-mismatch dynamics affected the survival of larval cod (*Gadus morhua*) populations from 1960 to 2002 and whether these processes vary between major spawning grounds.

Variability in fish recruitment has long been linked to the overlap in space and time between larval and juvenile fish and their prey [1]. Cushing's match-mismatch hypothesis [4], [5] proposes that recruitment variability is a consequence of starvation during the critical larval period. The hypothesis suggests that most fish in temperate waters spawn at a fixed time, while the prey of larval fish (zooplankton) depend on the variation in *time of onset* and *duration* of spring blooms [4], [5]. A match between prey and larval abundance can result in high larval fish growth rates and high survival. On the other hand, a mismatch can cause starvation, low growth and survival rates, and, consequently, low recruitment. The timing of peak prey abundance varies annually with the physical and biological conditions in the ocean. Years of strong overlap in the peak abundance of larval fish and their prey can substantially increase their larval survival probability [6]. However, the role of the duration of primary production in recruitment variability has not been well explored (but see [6]).

The timing, amplitude, and duration of spring blooms directly and indirectly affect the survival and recruitment of many marine species, particularly in temperate and boreal ecosystems (e.g. [6]). At higher latitudes, the onsets of spring blooms are largely determined by seasonal patterns of light, temperature, and nutrients in the water column [7]. In years when the water column is colder than usual, the spring blooms generally occur later, while in years when the water is unusually warm, the spring blooms occur earlier [8] (but see [9]) The elevated primary production observed during warm years is a result of earlier stratification, a prolonged growth season, and increased metabolic rates across functional groups and trophic levels [10].

However, primary production alone does not determine survival of larval cod. A number of additional biological and physical factors are also known to play an important role [2], [3]. For example, larval cod are visual hunters and depend on light to detect prey items. The concentration of particles in the water column may therefore reduce visibility, lowering the encounter rate between larvae and their prey. If larvae are able to find food, they can achieve higher growth rates. As they grow, the number of potential predators increases and then decreases in a dome-shaped relationship [11]. If the larvae have a perfect overlap in space and time with their prey, but the prey are all too small or too large, the larvae will still die from starvation or have strongly reduced growth rates, which lowers their chance of survival to recruitment [12]. Even when prey availability is low, larval cod mortality can still be low if larval predation rates are not high, which can happen in the early part of the season [13]. Later in the season, larval fish predators such as invertebrates (e.g. jellyfish) and vertebrates (e.g. planktivorous fish such as herring) increase their metabolism, abundance, and consumption following seasonal increases in ocean temperature, making it harder for the larvae to survive [13]. There are also a multitude of physical forces at varying scales that can influence the survival of larval fish including advection into prey-depleted areas [14] or through the dome-shaped relationship between prey encounter rate and turbulence [15].

We used a coupled biophysical model to determine how match-mismatch dynamics could affect larval cod growth and survival across the North Atlantic. Our analysis focused on four main Atlantic cod spawning sites in the North Atlantic: Georges Bank, the southwestern coast of Iceland, the North Sea, and Lofoten on the west coast of Norway. For each location, we selected the warmest and coldest years, based on the annual average temperature over the upper 50 m (or to the deepest depth if shallower) between 1960 and 2002. Time-series of ocean temperatures from these years for each location were then incorporated into an individual-based model (IBM) to estimate quantitative values for the rate of growth and survival of larval cod.

Because the highest mortality for cod occurs during the larval and juvenile stages, improved predictions of adult population dynamics depend on accurate predictions of larval mortality. Therefore, it is critical to obtain a better understanding of the mechanisms driving match-mismatch dynamics, and how changes, e.g. in the timing or duration of production at one trophic level, affect the next trophic level. Here, we used our biophysical model to quantify how changes in temperature propagate through primary and secondary production and subsequently affect the growth and survival of Atlantic cod larvae. We found that a prolonged period of primary production increased the number of larval fish that survived during the early life stages. The prolonged season tends to increase the duration of the overlap between larval and prey production, resulting in a cumulative higher number of survivors. In addition, we analyzed how light, temperature, and prey abundance varied across the study sites and found that the importance of match-mismatch increased with latitude. Match-mismatch dynamics are more important in areas where there is greater seasonal variation in light and primary production. Together these results provide important information on the mechanisms that regulate cod recruitment dynamics.

## Materials and Methods

The individual-based model is described following the overview, design concepts, and details (ODD) protocol [16].

### Purpose

We developed an individual-based model (IBM) to simulate the early life history events of larval Atlantic cod for 30 days from first feeding. The IBM was designed to capture the complex feeding behavior of larval cod under changing environmental conditions (e.g. light, temperature, turbulence). We used a mechanistic approach that incorporates the physical characteristics and biological properties of the larva and its surroundings to estimate their interactions. A benefit of this approach is that the model is applicable for any given geographic location and for different species. The feeding behavior module of the model includes components of encounter, approach (pursuit), and capture success, while the mortality component estimates larval mortality rates from predation by invertebrates and piscivores, as well as from starvation. The IBM was used in combination with realistic environmental data to explore how physical and biological properties of larval cod habitats across the North Atlantic change through the spawning season and between years, and to analyze how such changes affect the survival and growth during the early life stages.

### State variables and scales

**Figure 1:**
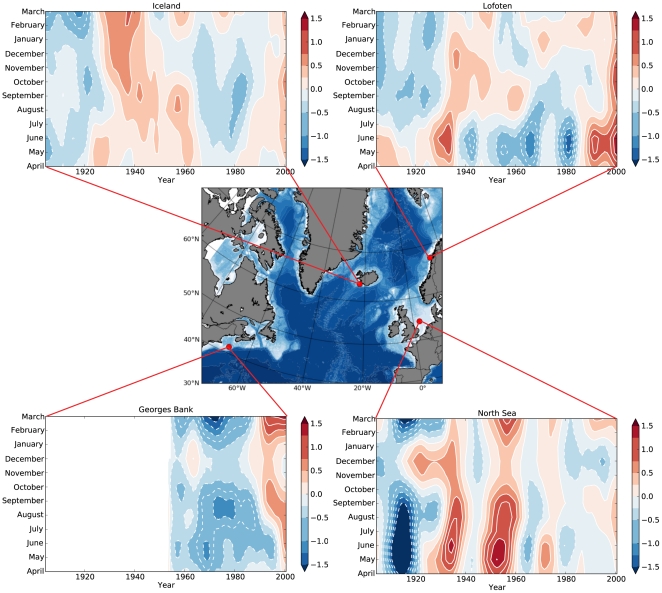
Map of four major spawning sites across the North Atlantic (middle) with time-series (1900–2006) of air surface temperature anomaly data for each site. Clockwise from top left Iceland, Lofoten, North Sea, and Georges Bank. The air temperature plots highlight the cold period during the 1960s and 1970s as well as the warm period during the 1930s and 1940s. Air surface temperature anomalies were calculated from a base climatology from 1961–1990, and smoothed using a 9-year running mean. The air temperature data [59] were provided by Nansen Centers in St. Petersburg (Russia) and Bergen (Norway) and are available from their web site at http://www.niersc.spb.ru/en/downloads/nansensat/nansensat/.

We assumed the size of the individuals at first feeding was 5±0.5 mm and that each cohort consisted of 100 individuals. We simulated the growth and survival of each individual of a cohort for 30 days from first feeding. The first release of a cohort was assumed to take place on January 15 with additional releases every 15 days for the rest of the year. We allowed the larvae to be released into the water column throughout the year to analyze how cold and warm years may allow for survival outside of the typical observed spawning periods. One motivation for this was the observed changes during the strong warming in the 1930s when cod spawned during autumn much more than what is common today [17]. We repeated these simulations for the average coldest and the warmest year (Table 1) between 1960 and 2002 at four locations in the North Atlantic. Each location represents a historically important spawning ground for Atlantic cod (Fig. 1). The ocean environment was represented in our model using a model reanalysis of historical ocean temperature and surface wind stress [18], and modeled light and ocean turbulence. Observed and averaged chlorophyll concentrations from satellite data (http://seadas.gsfc.nasa.gov/) combined with ocean temperature data were used to calculate a proxy for the zooplankton concentration. Modeled predation from both vertebrates (e.g. fish) and invertebrates (e.g. jelly fish) were included (Text S1). The numbers of predators in the water columns were assumed constant with depth and time; however, predation pressure changed as a function of light level and larval size.

**Table 1:** Cold and warm years used in the simulations to estimate growth, survival, and fitness at the 4 spawning locations in the North Atlantic.

Station	Minimum (Cold)	Maximum (Warm)
Georges Bank	1965	1999
North Sea	1963	1999
Iceland	1983	1960
Lofoten	1977	1990

### Process overview and scheduling

For every time step of the mechanistic model, we calculated a sequence of processes. The outcome of these processes determined the behavior of the larva given the current surroundings such as prey concentration and light level at a given depth. The IBM calculated each process (such as swimming speed, visual ability, encounter rate) based on the current physical and biological conditions at the depth where the larva was situated for the given time of day, year, and geographical location. The IBM simulated the feeding behavior in a sequential manner: prey encounter, approach, capture, and ingestion. For each time-step, ingested biomass was stored in the stomach and used for metabolic demands and for growth, which were calculated as separate components. If the ingested biomass adequately satisfied the requirement to sustain maximum growth rate, growth rate was defined to be strictly temperature limited (food unlimited) [19]; otherwise the growth was food limited [20].

### Design concepts

#### Emergent properties

The overall population structure was revealed through the average of the properties of the individuals. This included the depth distribution, survival probability, fitness (survival rate times weight), and growth rate for different cohorts of fish spawned throughout the year at the four selected spawning locations in the North Atlantic.

#### Sensing

To allow for diel vertical migration the larvae could optimize their depth location by balancing the probability of mortality against ingestion rates above and below their immediate depth position [21]. The vertical distance up and down they could “sense” was defined by how far they were able to swim (routine swimming speed) within the duration of one time step [22]. The individual larvae were allowed to swim to the depth level that maximized ingestion but minimized mortality rates. This trade-off between mortality and ingestion rates represents the ability of the larva to optimize its phenotypic plasticity to the environment [21]. From this behavior emerged a diel vertical migration pattern. This type of cognitive decision-making has been observed in other modeling studies [23] and experiments [24] and is believed to be connected to the fishes ability to smell, taste and see [25].

#### Interaction

This fish foraging did not have any influence or feedback on the prey concentration in the model and there was no interaction (e.g. cannibalism, competition) among individuals within a cohort or between cohorts, thus only density-independent processes were included.

#### Stochasticity

To simulate the escape mechanism of zooplankton they were assumed to jump/move away from the approaching predator at a random angle ±30 degrees from the fish gape [26]. The initial larval size distribution was randomly selected from a normal distribution that varied ±10% around 5 mm.

#### Model data

For each time step we recorded the state variables (length, weight, stomach content) of each individual of each cohort together with the properties of the environment (temperature, depth position, prey concentration, light levels, turbulence) that the individual fish experienced. The combined recorded information for all individuals was combined to derive properties and characteristic at the population level.

### Initialization

Each individual larva was released at standard length (*SL*) of 5.0±0.5 mm (90 µg, dry-weight) and with 30% full stomach to ensure the larvae had energy enough to meet metabolic demands for the first time-step. All individuals were released at 15 m depth and allowed to swim and behave within the water column.

### Input

**Figure 2:**
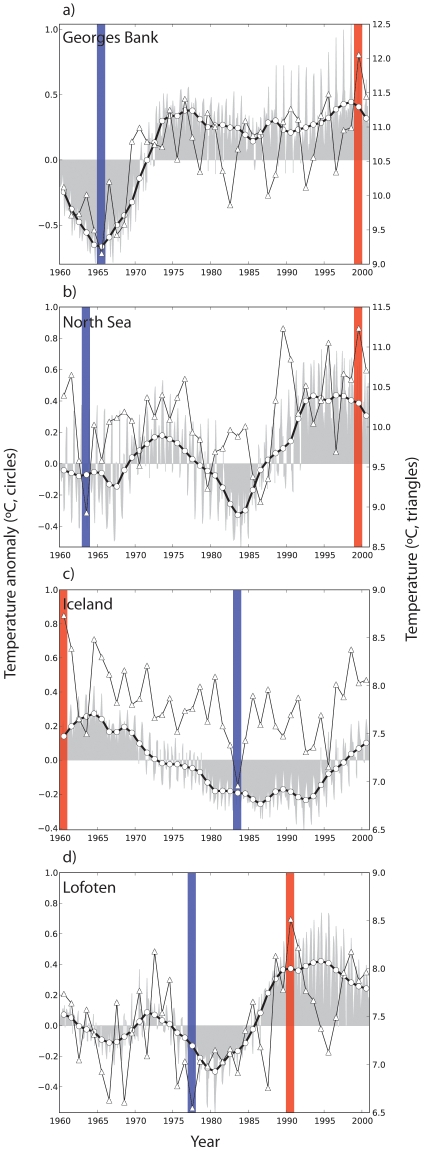
Time series of surface temperature reveal both colder (e.g. 1960's) and warmer (e.g. 1990's) time periods relative to the climatology (1961–1990). The red bar indicates the warmest year on average for the upper 50 meters (or to the deepest depth if shallower) while the blue bars indicate the coldest. *Surface temperature anomalies* (°C left y-axis, black thick line with circles) were smoothed with a 9-year running mean, and plotted with the unsmoothed *surface temperature anomalies* (°C, left y-axis, light grey), and *surface temperature* (°C, right y-axis, dark grey lines with light triangles). All time series are shown from 1960 to 2002 for the four studied locations in the North Atlantic.

To adequately describe realistic environmental properties at each of the four spawning grounds (Fig. 1) we used the Simple Ocean Data Assimilation (SODA, http://www.atmos.umd.edu/~ocean/) database. SODA is a global re-analysis of the ocean climate [18] for the period 1958 to 2002 based on an ocean model with a resolution of 0.5°×0.5° latitude-longitude. The model uses assimilation to constrain simulations to observed temperatures and salinities, which were derived principally from the World Ocean Atlas [27]. For each spawning ground (defined by a single latitude-longitude position), we created a time-series of temperature, salinity, and u and v surface wind stress by spatially interpolating the four surrounding SODA grid points. The result was a time series (1960–2002, 5-day temporal resolution) of temperature for all depths at each spawning ground that we used as input to the IBM. Once read into the IBM, the SODA data were interpolated both temporally and spatially to the larval depth position for the specific time of year. Based on the temperature time-series we also calculated the temperature climatology 1961–1990. This climatology was used to estimate ocean temperature anomalies (Fig. 2), which were further used to modulate the prey concentrations. The u and v surface wind stresses were used to estimate the turbulence level at the depth of each larva using a functional relationship [28].

**Figure 3:**
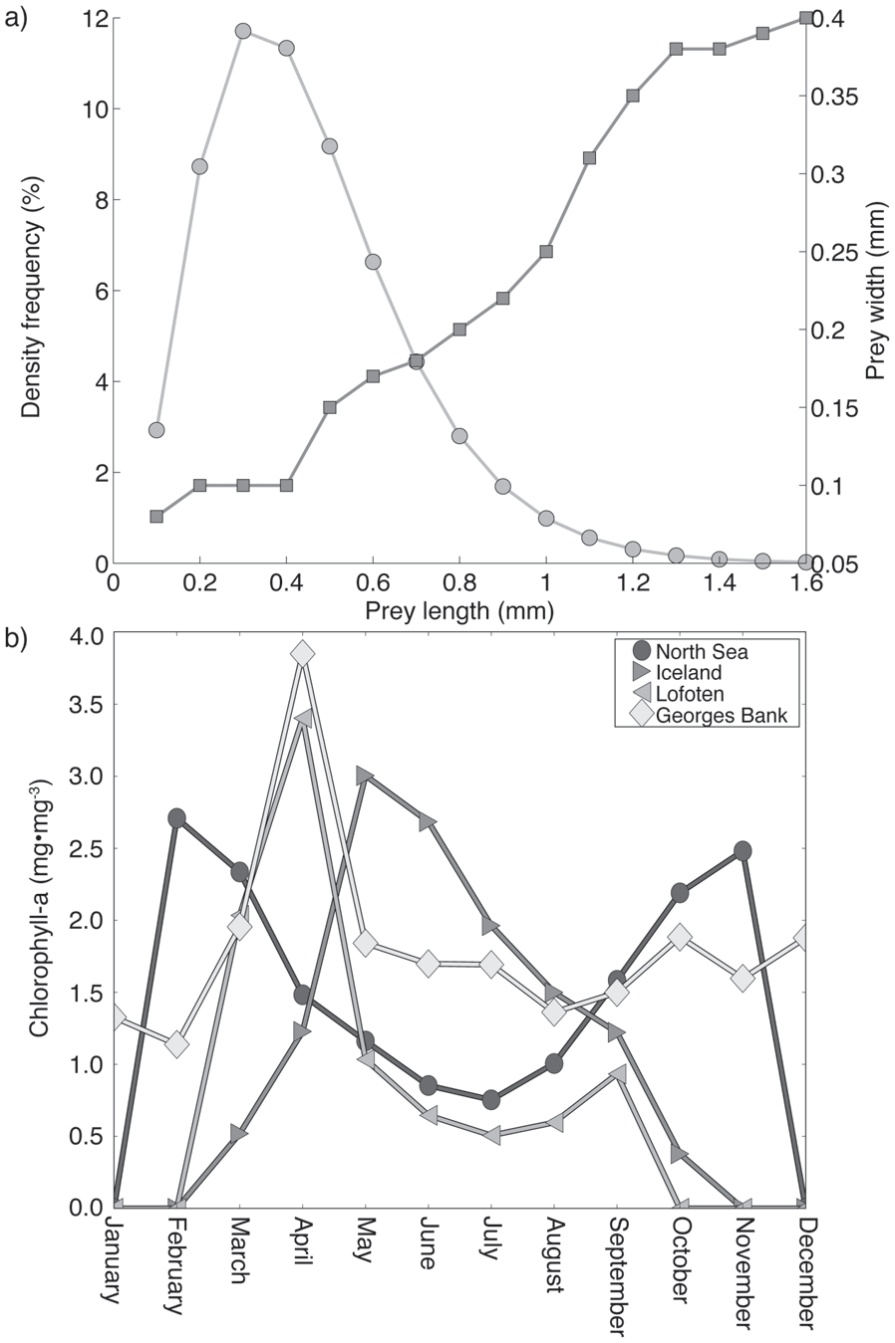
Frequency distribution of modeled prey sizes and observed monthly concentrations of chlorophyll-a. The relationship between prey length and prey size groups used in the model as suggested in [34] are shown in a). No size interval contains more than 12% of the total abundance (circles). The squares show the relationship between width and length [33] of the prey items, which is essential for estimating prey image size, and visibility to the larval cod. b) Climatological chlorophyll-a (mg•m^−3^) values from January to December for the North Sea, Iceland, Lofoten, and Georges Bank stations (also see Fig. 1). Chlorophyll-a values are used together with the temperature anomaly data to calculate the monthly prey (mesozooplankton) concentration.

A global atlas of monthly (January to December) average (1998–2008) chlorophyll-a values were obtained from the SeaWiFS project website (http://seadas.gsfc.nasa.gov/). Chlorophyll values from the nearest four grid points surrounding the spawning locations were interpolated in space and time and used to create time-series of chlorophyll-a values. Light was modeled as a function of day of the year, latitude, and depth [29], while the attenuation coefficient was modeled as a function of monthly climatologically chlorophyll-a values (Text S1). The climatology of chlorophyll-a was used to estimate the climatology of the seasonal variation of zooplankton abundance. A lack of data prevented using actual time-series of zooplankton for all of the study locations, thus the use of the chlorophyll-a values as a proxy for the seasonal variation in zooplankton as suggested in the literature (e.g. [30]). Annual and inter-annual variability in zooplankton abundance was included through temperature, as the productivity in the ocean changes with temperature [31]. Warmer years tend to result in higher production while colder years result in lower production [8], [32]. Consequently, we used the monthly temperature anomaly to estimate monthly anomaly in zooplankton concentration. These were then interpolated to daily values, which were then added to the climatological zooplankton concentration for those days. The scaling was determined from literature reviews and comparison between the zooplankton production in warm and cold years (e.g. [33]), which suggest the maximum zooplankton production anomaly is approximately 50% of the mean seasonal zooplankton variability. For each site the mean maximum zooplankton concentration for the year was set to 80 prey items per liter, therefore the minimum and maximum zooplankton concentrations between the coldest and warmest years ranged between 0–120 prey items per liter. The prey was divided into size intervals of 100 µm ranging from 100 to 1600 µm according to the algorithm described in [34] (Fig. 3a). This range includes the typical size range (length and width) of *Pseudocalanus* and *Calanus finmarchicus*, the main prey species for cod larvae found in the four locations. In both cold and warm years the larvae usually have a relatively high number of prey items available to feed on, and the estimated numbers of prey have been compared to observations on Georges Bank and are within the observed ranges (see Text S1 for details).

To analyze whether our modeling results are seen in nature we correlated observations of juvenile abundance (0-group, age 5–6 months) with number of recruits (age 1 for North Sea and Georges Bank, and age 3 for Iceland and Lofoten). Cod recruitment and juvenile (5–6 months) data were obtained from ICES (http://www.ices.dk/datacentre/StdGraphDB.asp).

### Model assumptions

We had to make some necessary assumptions in order to use consistent data and methods across all our study locations. For example, we assumed that chlorophyll-a values can represent the timing of zooplankton in the water column. Previous work has showed that this is likely a valid assumption [6], although the development of zooplankton bloom may also depend on a number of other factors not included in our model. The estimated zooplankton in the model varied seasonally with the variation in chlorophyll-a values, which means that the peak abundance occurred during spring bloom and a secondary peak occurred in early autumn. The timing and seasonality differed across the four study sites.

The model captures the most important features of the developing feeding behavior and development of larval cod during their first 30 days [20], [21]. Our work represents a new approach to understand the non-linear response of fish survival to physical and biological forcing, although not all factors important to larval fish growth and survival were included in the model.

Details on the different components of the IBM and the model runs are available as supporting information (Text S1).

## Results

**Figure 4:**
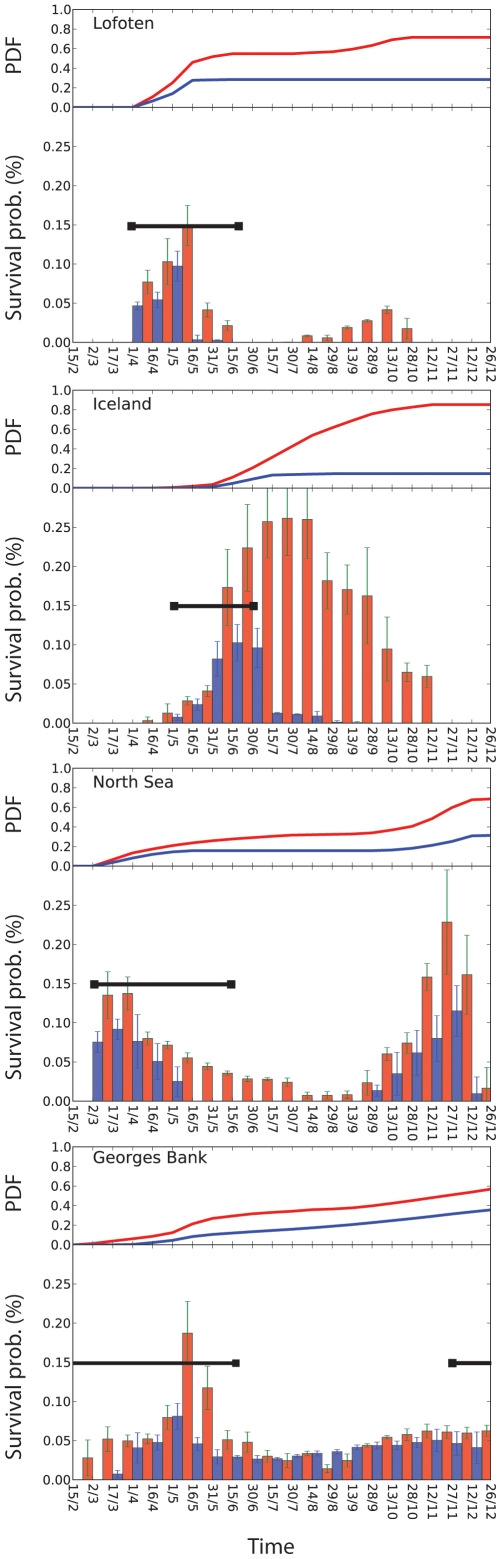
Average and cumulative survival probabilities. The upper panel shows the probability density function (PDF) of cumulative survival through the year. The slope of the PDF indicates the time period of importance to the cumulative yearly survival. In Lofoten, a short time window during spring accrues most of the cumulative survival during the year, while on Georges Bank, the slope is more continuous suggesting that a longer time window when high growth and survival rates are possible. The lower panel shows the average probability (%) of larval cod survival from hatching to age 30 days in warm (red) and cold (blue) years at four different spawning locations in the North Atlantic. The black thick lines indicate the time-period when larval fish 0–30 days old are observed in the water column.

A combination of temperature, light and prey concentrations created an optimal temporal window for survival (Fig. 4). The timing and duration of this optimal window differs strongly between spawning locations in the North Atlantic, with the shortest optimal window in the north (e.g. Lofoten), and the longest in the south (e.g. Georges Bank). In general, medium to high larval growth rates were achieved when day-length exceeded 10 hours (i.e. March to September, Fig. S1), a level that enables continuous feeding during the day and provides more energy stores for growth. The period during the year available for larval cod to sustain high growth rates widens increasingly away from higher latitudes. In Lofoten the day-length available for feeding varies from 0 to 24 hours while on Georges Bank the day-length is more constant throughout the year varying from 10 to 17 hours available for feeding (Fig. S1). We also calculated the combined effect of survival and weight at age every 30 days by multiplying the state variables. The pattern of fitness is identical to the survival pattern so only survival is shown.

Positive water temperature anomalies increased growth rates, which are beneficial for survival (Fig. 4) and fitness (not shown) at all spawning locations. Within the time-period when larval fish were observed in the water column (Fig. 4), modeled survival was 175%, 154%, 385%, and 308% higher in a warm versus cold year for Lofoten, North Sea, Iceland, and Georges Bank cod stocks, respectively. Modeled average survival probability (%) at age 30 days and standard error in cold (warm) years when larval fish were observed to be present were 0.03±0.015 (0.07±0.02), 0.07±0.009 (0.14±0.017), 0.03±0.016 (0.06±0.03), 0.05±0.013 (0.08±0.014) for Lofoten, Georges Bank, Iceland, and the North Sea, respectively. Elevated ocean temperatures allowed for an extended time-period when the conditions in the water column facilitated high larval survival. Conversely, chances of survival were lower in a cold year, where the time-period of adequate conditions in the larval environment was much shorter (Fig. 4). The prolonged productivity of a warm year resulted in an integrated growth potential and survival that far exceeded the potential in a cold year, although for any individual 30-day period the growth and survival do not differ substantially between warm and cold years.

**Figure 5:**
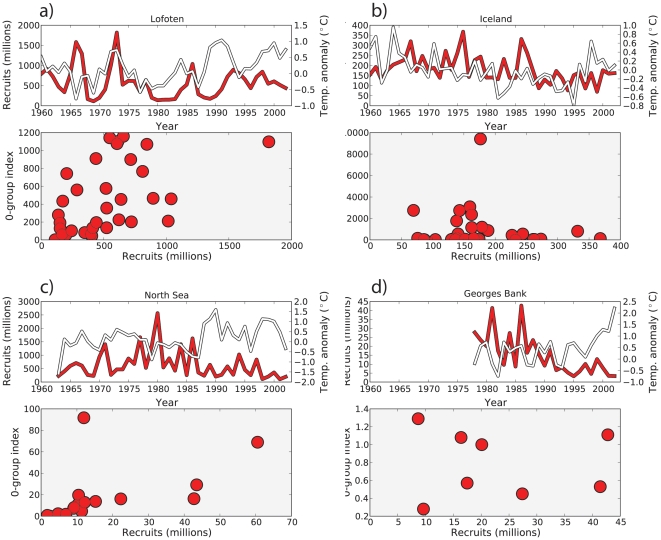
The relationship between number of recruits (millions), temperature anomalies, and 0-group index (index for number of juveniles). Upper panel shows number of recruits (red line, left axis) and temperature anomalies (white, right axis) as a function of year for a) Lofoten, b) Iceland, c) North Sea, and d) Georges Bank respectively. The lower panel shows a scatter plot of the number of recruits versus 0-group index (5–6 months old).

To analyze the importance of survival through the larval and juvenile stages on recruitment we quantified the relationship between empirical data on the number of recruits with data on the number of juveniles. The correlation coefficents between observations of juvenile abundance (0-group index, 5–6 months) with recruitment were r = 0.53 (*p* = 0.09) and 0.52 (*p* = 0.0016) for the North Sea and Lofoten respectively, while Iceland and Georges Bank were −0.08 (*p* = 0.32) and −0.05 (*p* = 0.89) respectively. Temperature conditions during the larval and juvenile stages can have a strong influence on the number of individuals that survive to recruitment. To analyse how environmental conditions during larval and juvenile stages affect survival to recruitment, correlations were calculated between temperature and number of recruits. The temperature was lagged by 1 year for the North Sea and Georges Bank and 3 years (age at recruitment) for Lofoten, and Iceland. The correlation coefficients were r = −0.39 (*p* = 0.01), 0.14 (*p* = 0.35), 0.29 (*p* = 0.05), and 0.01 (*p* = 0.96) for the North Sea, Iceland, Lofoten, and Georges Bank, respectively. Finally, we calculated correlations between juveniles and temperature and found r = −0.03 (*p* = 0.92) , −0.08 (*p* = 0.65), 0.48 (*p* = 0.002), 0.38 (*p* = 0.22) for the North Sea, Iceland, Lofoten, and Georges Bank, respectively (Fig. 5).

## Discussion

The match-mismatch hypothesis is usually associated with the importance of the timing of larval fish hatching and peak prey abundance [1], [4]. Our results indicate that the cumulative growth and survival over the entire spawning period is critical [4] in determining year-class strength. In other words, there does not have to be a perfect match between time of hatching and peak abundance of prey items as long as the water column contains sufficient prey and favorable physical conditions. The relative importance of match-mismatch dynamics changes with latitude as light, temperature, and food conditions may limit the time period during the year when high feeding, growth, and survival are possible.

**Figure 6:**
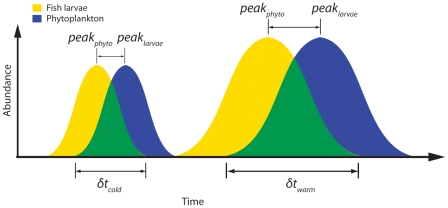
Conceptual diagram of the temporal relationship between larval fish abundance (yellow) and phytoplankton biomass (blue). During the time period when these overlap (green), the larvae will have adequate food conditions and therefore enhanced survival probability. In years when the water temperature is warmer, this time-period (*duration*) is longer compared to in colder years. In addition, during years of high prey abundance (right), the consequences of the lag in time between the occurrence of peak food and peak larval production is less important. Increase in primary production in warmer years enables higher total production over a longer time window because production starts earlier in spring. Based on Figure 1 in [6].

Our modeling results also suggest warmer-than-average ocean temperatures slightly enhance survival through the first 30 days, because warmer ocean temperatures create a longer period of high biological productivity and facilitate high growth rates and survival. During warm years when total primary production is high, the match between peak prey and larval abundance is even less important than the cumulative effects of growth and survival because of the longer period of adequate conditions for survival and growth (Fig. 6). With higher growth rates, larval fish may escape predation later in the year and cumulative survival can result in a stronger year-class.

### Cumulative survival of individuals through the spawning season is a key determinant of year-class strength

Our results provide an explanation of the underlying mechanisms of the match-mismatch hypothesis. The duration of overlap in space and time between the abundance of larval fish and prey increased the cumulative survival over the year (Fig. 4). When ocean temperatures are above average, the optimal period when the necessary physical and biological properties are present is longer, resulting in higher larval growth rates and survival (Fig. 4). A temporal match between the timing of larval hatching and peak prey abundance may be beneficial for a strong year-class, but it is not required. In fact, a combination of average prey concentrations (5–15 prey⋅L^−1^), enough light (>12 hours d^−1^) in the water column to allow for successful foraging, and above average water temperatures can lead to high survival through the larval stage. Given such environmental conditions, larval fish can hatch and survive over a longer period, possibly producing a strong year-class even when prey abundance is average. Total prey availability over the spawning period is more important than the temporal overlap between peak prey and larval production (Fig. 5, 6).

Our results also suggest that warm years produce a cumulatively high number of larval survivors, which can lead to high recruitment [35]. Although we only considered the first 30 days after hatching, year-class strength is not defined by the end of that stage and mortality may still be high throughout the first year after hatching [36], [37], [38]. Nonetheless, the first 30 days are a critical phase when rapid development reduces mortality by predation. To examine the importance of survival through the larval and juvenile stages on recruitment we analyzed the correlation between the number of recruits and 5–6 month old juveniles. High correlations between juveniles and recruitment were found for the North Sea and Lofoten regions. In Iceland and Georges Bank, correlations were not signficant (Fig. 5). We also found that environmental conditions during the larval and juvenile stages had a significant effect on the number of recruits for the North Sea and Lofoten, while for Iceland and Georges Bank the correlations were not significant.

The correlations found in this study are consistent with previous work by Planque and Frédou [39], which showed that increased temperatures were associated with above-average recruitment for stocks in the low-range (≤6°C) bottom temperature habitats (e.g. Lofoten), and below-average recruitment to stocks in the high-range (≥9°C) habitats (e.g. North Sea). For the stocks in the temperature middle-range (7–8°C) habitats (e.g. Georges Bank and Iceland) temperature changes had seemingly no impact on recruitment. The weaker correlations for Iceland and Georges Bank suggests other physical and biological processes there may control survival of cod after the larval and juvenile stages [2], [3].

The lack of correlation in the North Sea is likely driven by changes in prey species composition [12], which we were not able to include in the model. In accordance with Planque and Frédou [39] we found a negative correlation between observations of temperature and recruitment of r = −0.39 in the North Sea, and positive correlation for Lofoten r = 0.29. However, our model suggested a positive effect of increased temperature on survival probability for the North Sea cod. One possible reason for the discrepancy between model and empirical observations is because we did not include the shift in species composition between *C. finmarchicus* and *C. heloglandicus* that occurs in the North Sea during extended warm periods [12]. During warm years in the North Sea there is less advection of *C. finmarchicus* rich water from the Norwegian Sea [40] and *C. finmarchicus* are replaced with *C. helgolandicus* from the south [12]. *C. helgolandicus* have a lower lipid content, a different phenology, and smaller size compared to *C. finmarchicus* [12], [41]. *C. helgolandicus* spawn later in the year compared to *C. finmarchicus*, which creates a mismatch in the timing and presence of larval cod and prey items in the North Sea [42]. In addition, since *C. helgolandicus* are smaller than *C. finmarchicus*, cod larvae would have to encounter more prey items during the same time period to sustain growth. Therefore, warming results in lower caloric food sources for larval cod in the North Sea and a subsequent reduction in larval survival and recruitment. We were not able to include such a prey shift in the model because of the complexity of the implementation. Instead we focused on comparing habitats across the Atlantic with the intention of understanding the underlying mechanisms of larval cod survival in cold and warm years. Even with modeling limitations, our work begins to disentangle which processes are important.

### “Bigger is better”

The transition from small larval fish with limited possibilities for locomotive response to escape from predators into opportunistic foragers occurs faster when growth rates are high. Growth rates in warm years reduce the transition time from larval to juvenile, leading to a shorter time span in the larval stage. Faster development rates reduce larval vulnerability to predators because invertebrate predation in the model is size-dependent [43]. Metabolic rate, regulated by body size and temperature, is the fundamental constraint of ecological processes in the marine realm [44]. As the water temperature increases, larval cod and their prey grow and develop faster. These conditions enable cod to sustain energy demands from increased metabolic rates by feeding on increasingly larger prey items [45]. With greater food resources, larval fish can grow faster. Since the speed of larval development is related to their size, not their age [46], faster growth ensures that larvae develop faster. Several studies suggest that in a cohort of larval fish of the same age, but different sizes, the larger individuals have a greater chance of survival, and consequently a higher recruitment potential [47]. This pattern is known as the “bigger is better” hypothesis (see [2]) and suggests that fast growth is a precondition for high survival.

Larval fish can increase their size 10-fold during their first 30 days, if they are able to find food, making this period critical for determining survival [38]. As larval fish pass through the developmental stages before they reach metamorphosis, greater size increases their motility, helping them avoid predation [48]. Larger larvae are also able to feed on a wider variety of prey sizes, which makes them less susceptible to changes in prey species composition [12]. Access to a wider range of food resources results in a positive feedback that enables larval fish to maintain increased metabolic demands when ocean temperatures are warmer and obtain the required food to sustain high growth rates. However, a shift to warmer waters also affects the production of prey for larval fish. In fact, a change in temperature may lead to a switch in the community structure and a change in the dominant species of an ecosystem [49], with possible consequences of a more limited, less nutrient rich, prey distribution for cod [50].

### Latitudinal variation in the importance of match-mismatch

The relative importance of match-mismatch differed across our study sites. The latitudinal variation in the timing and length of spring blooms [51] results in major differences in phytoplankton availability throughout the year [51]. Differences between light and nutrients across the North Atlantic cause match-mismatch dynamics to become increasingly more important toward higher latitudes as the period available for phytoplankton and zooplankton blooms decreases. At the same time, the lower variation in light level and biological productivity in the lower latitudes likely moderates the importance of match-mismatch [31], [52]. In Lofoten and Iceland, survival probabilities (Fig. 4) are clearly limited by how the light (Fig. S1) controls the timing of productivity. Because of the short growing season in northern regions, it becomes increasingly important for larval fish to hatch as early in the season as possible in synchrony with their food and take advantage of the full growing season. A temporal and spatial match with larval and food production extends the duration of high growth and survival, which results in a strong cumulative survival. A warm year will be beneficial at northern latitudes, as the onset of the spring bloom will occur early, allowing for a maximized duration when larval fish and their prey are present in the water column. This overlap allows for increased survival of larval cod that hatch early in the season compared with a cold year when these individuals would usually die from starvation. As you move south in the North Atlantic, the seasonal variation in light and day-length decreases, enabling primary production to continue for a longer portion of the year. Larval food production in southern areas is much less variable through the year compared to Lofoten where food is available only within a limited time window. Therefore, on Georges Bank and in the North Sea, the modeled survival rates through the year are much less variable because of the more consistent availability of light and food (Fig. 3b, S1).

Our results are dependent on the modeled prey availability, which can differ from observed availability. At some sites, the model predicts potentially high survival rates during periods when there has been no observed major spawning, e.g. in Lofoten and Iceland in the fall. Larval survival could potentially be high during these periods as predicted, but survival at the juvenile and later stages for individuals that hatch in late autumn are probably very small due to observed low food resources during these times (e.g. [42]). Light, temperature, and prey are favorable during the 30-day modeling window, but may be less optimal beyond it. In Lofoten, the model suggests that October could be a time for autumn spawning. However, the key prey item *C. finmarchicus* is already in diapause by October [53], and the prey life cycle is not included in the model. In the North Sea, the latter part of the autumn is suggested as a potentially high survival time. In earlier years, cod have been observed to spawn during autumn in the northern North Sea [54]. On Georges Bank, the model also predicts that late autumn spawning could be beneficial for larval survival and spawning is observed in this region during November [55]. Typically, predicted larval survival outside of the usual spawning season occurs during warm years when productivity is high. Indeed, during the strong warming in the 1930s, cod did spawn during autumn much more than what is common today [17]. This was probably due to combination of greater ecosystem productivity [17] and more older spawning age classes [56], which spawned earlier in the season than younger age classes [57].

### Conclusions

Current estimates suggest that 85% of the world's fish populations are fully exploited, overexploited, depleted, or recovering [58]. The North Atlantic cod support one of the most important commercial fisheries with an estimated value of $1.625 billion per year. Year-to–year variation in the number of cod in the ocean has large socio-economic consequences. The negative effects of this variability could be better managed if we understand and are able to predict variations in fish production. For decades there has been a strong emphasis on how year-class strength is related to the match-mismatch between predator and prey, or the timing of peak larval fish and prey abundance [4]. Our results provide a mechanistic understanding of the factors driving strong recruitment for North Atlantic cod. We found that survival rates of larval fish are dependent not only on a single episodic peak overlap between prey and larval fish, but also on the duration of the overlap. In fact, the duration is probably more important to the overall survival of larval cod than timing of peak production of larval cod and prey. During warm years, an extended time-period increases the seasonal cumulative survival of larval cod in northern regions (Fig. 6). Warm years lead to higher productivity, faster growth rates and increased probability of survival. By integrating physical and biological factors in predicting survival in early life stages, we can more accurately understand the population dynamics of commercial fisheries. Our combined empirical and modeling approach can be applied to other ecosystems and taxa to resolve ongoing questions regarding variability in species abundance.

## Supporting Information

Figure S1 **Day-length variation as a function of geographical location and time of the year.** Day-length shown at the surface (a) and at 20 meters depth (b) for four locations in the North Atlantic: Georges Bank, North Sea, Iceland, and Lofoten.
(TIF)

Text S1 **Detailed methodology for individual-based model.**
(DOC)
