# Supplementary figures and images for: Recruitment Variability in North Atlantic Cod and Match-Mismatch Dynamics
Source: PLoS One. 2011 Mar 7;6(3):e17456. doi: 10.1371/journal.pone.0017456 (PMC3049760; doi:10.1371/journal.pone.0017456)

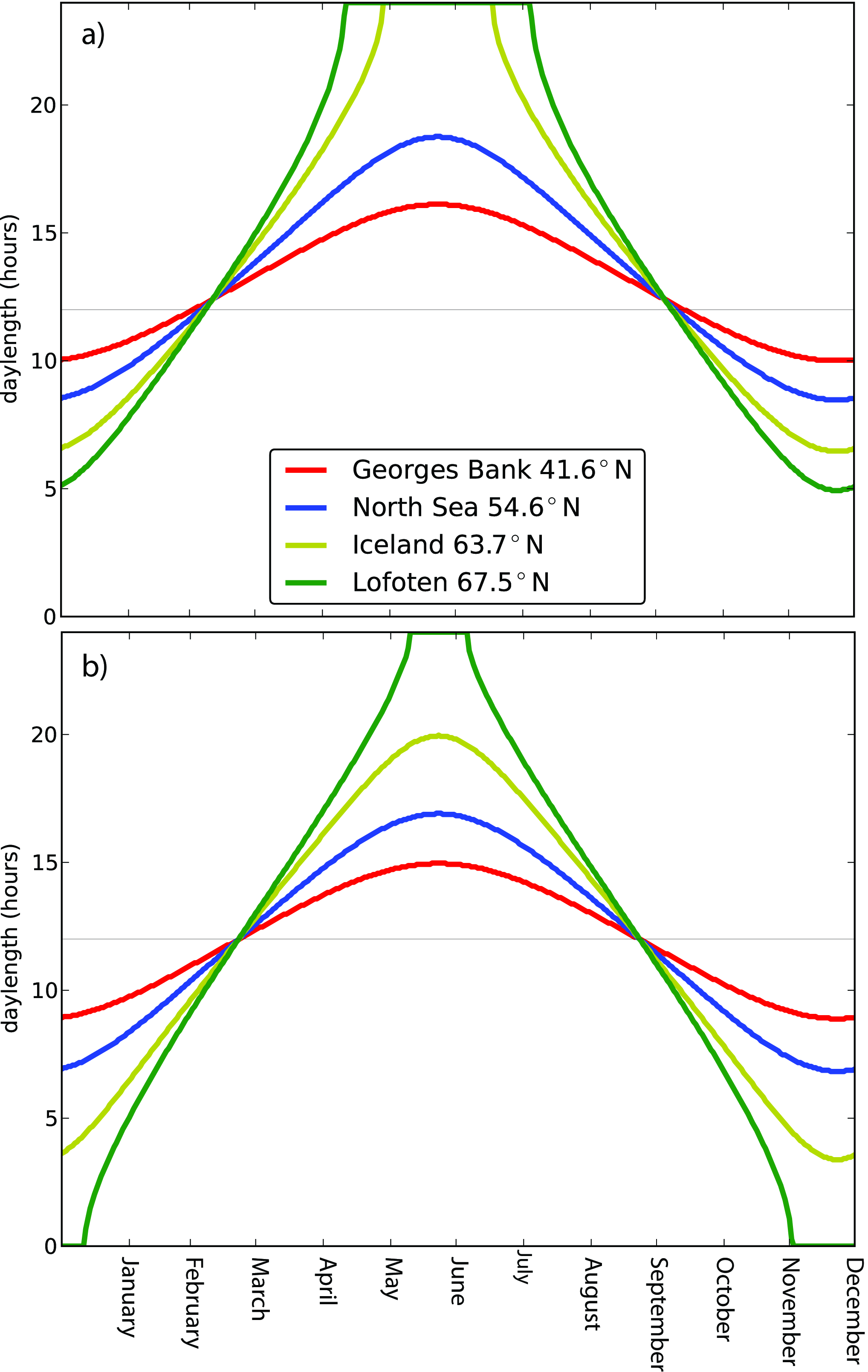

Supplement: Figure S1 — Day-length variation as a function of geographical location and time of the year. Day-length shown at the surface (a) and at 20 meters depth (b) for four locations in the North Atlantic: Georges Bank, North Sea, Iceland, and Lofoten. (TIF) [file pone.0017456.s001.tif]
