# Supplementary material for: Recruitment Variability in North Atlantic Cod and Match-Mismatch Dynamics
Source: PLoS One. 2011 Mar 7;6(3):e17456. doi: 10.1371/journal.pone.0017456 (PMC3049760; doi:10.1371/journal.pone.0017456)
Supplement: Text S1 — Detailed methodology for individual-based model. (DOC) [file pone.0017456.s002.doc]

**Text S1**

**Supporting information**

***Submodels***

Detailed descriptions of the individual-based model (IBM) have been published previously [1,2,3], but here we have added new methods on the calculation of prey concentrations and attenuation coefficients. The IBM consists of a number of models including the mechanistic feeding model, stomach and growth model, mortality model, and larval vertical behavior model.

To adequately describe realistic environmental properties at each of the four spawning grounds (Fig. 1) we used the Simple Ocean Data Assimilation (SODA, <http://www.atmos.umd.edu/~ocean/>) database. SODA is a global re-analysis of the ocean climate [4] for the period 1958 to 2002 based on an ocean model with a resolution of 0.5°x0.5° latitude-longitude. The SODA model uses assimilation to constrain simulations to observed temperatures and salinities, which were derived principally from the World Ocean Atlas [5]. For each spawning ground (defined by a single latitude-longitude position), we created a time-series of temperature, salinity, and u and v surface wind stress by spatially interpolating the four surrounding SODA grid points. One result was a time series (1960-2002, 5-day temporal resolution) for all depths at each spawning ground that we used as input to the IBM. Once read into the IBM, the SODA data were interpolated both temporally and spatially to the larval depth position for the specific time of year. Based on the temperature time-series we also calculated the temperature climatology 1961-1990, which was used to estimate ocean temperature anomalies.

**Feeding model**

Feeding was estimated every time-step (1 hour) in three sequential steps; calculation of (*i*) prey encounter, (*ii*) prey approach-pursuit, and (*iii*) capture.

(*i*) Pause-travel [6,7] feeders such as larval cod visually search for prey during the pause phase. During pause, the encounter rate between larva and prey was assumed to depend on the prey concentration, the visual ability of the larva [8], and the turbulence level. Larval visual perception is a function of larval development (size) and light level. Light level was calculated as a function of hour of the day *h* and year, depth *z*, and the diffuse light attenuation (*k*, water clarity) [9]. The diffuse light attenuation coefficient was calculated as a function of the chlorophyll-a value according to the formula in [10]:

$k\text{=}k_{0}+0.054\cdot Chla^{2/3}+0.0088\cdot Chla$ ()

where, *k*_0_=0.1 when chlorophyll-a (*Chla*, mg•m^-3^) is zero.

A global atlas of monthly (January to December) average (1998-2008) chlorophyll-a values were obtained from the SeaWiFS project website (<http://seadas.gsfc.nasa.gov/>). Chlorophyll values from the nearest four grid points surrounding the spawning locations were interpolated in space and time and used to create time-series of chlorophyll-a values. The climatology of chlorophyll-a is used to estimate the climatology of the seasonal variation of zooplankton abundance. Annual and inter-annual variability in zooplankton abundance is included through temperature, as the production in the ocean changes with temperature [11]. Warmer years tend to result in higher production while colder years result in lower production [12,13]. Consequently, we used the monthly temperature anomaly to estimate monthly anomaly in zooplankton concentration. These were interpolated to daily values and then added to the climatological zooplankton concentration for those days. The scaling was determined from literature reviews and comparison between the zooplankton production in warm and cold years (e.g. [14]), which suggest the maximum zooplankton production anomaly is approximately 50% of the mean seasonal zooplankton variability. For each site the mean maximum zooplankton concentration for the year was set to 80 prey items per liter, therefore the minimum and maximum zooplankton concentrations between the coldest and warmest years ranged between 0 – 120 prey items per liter. The prey was divided into size intervals of 100 μm ranging from 100 to 1600 μm according to the algorithm described in [15] (Fig. 3a). This range includes the typical size range (length and width) of *Pseudocalanus* and *Calanus finmarchicus*, the main prey species for cod larvae found in the four locations*.* In both cold and warm years the larvae usually have a relatively high numbers of prey items available to feed on, and the estimated numbers of prey have been compared to observations on Georges Bank and are within the observed ranges.

The visual perception of a prey item at a given depth was estimated as a function of the attenuation coefficient (clarity of water), beam attenuation coefficient (the loss of light between predator and prey at the same depth level over their separation distance), prey contrast, and image area. In addition, turbulence affected the number of prey swept into the perception area of the larvae during pause [1]. During low to medium turbulence, the pursuit and attack speed of the larva may exceed the turbulent movement of the prey, and the larva may benefit from turbulent flow of prey into its visual sphere. During high turbulence, the flow of prey in and out of the visual sphere is too fast for the larva to capture the prey items, which may be swept out of the visual detection sphere of the larva [16]. Prey were also allowed to swim into the larva’s perception area [17] during pause and search.

(*ii*) Assuming a prey was detected within the visual half-sphere, the larva had to move closer (pursue) to its prey and reach attack position without eliciting an escape response from the prey. This could only be achieved if the larva moved slowly and did not exceed the swimming speed that would create a deformation wave in front of the larva, which would alarm the prey [18]. The larva was assumed to be successful in the pursuit as long as distance from the larva to the prey (distance of perception) could be traveled in less than 10 s (average value from laboratory experiments [1]). If this criteria was not fulfilled the approach failed. Failed approach occurred when a larva detected a large prey (strongly visible) at a great distance outside of pursuit distance. Prey items did not have explicit behavior except the jump escape speed, which was proportional to prey length. A successful pursuit enabled the larva to reach attack position.

(*iii*) A successful attack depended on the relative size ratio between the prey and the predator, the gape size (larva cannot swallow a prey that has a width wider than its gape size), the attack speed of the larva, and the random jump angle of the prey [1,19]. An attack by the larva initiated an escape jump by the prey. A successful escape occurred if the prey jumped to a safe location outside of the view area of the larva. If the prey jumped out of the range of the larva, although still within the visible half-sphere of the predator, another attack would be initiated by the larva. We limited the maximum number of attacks to three. The probability of successful attack was determined using the function defined in [3].

**Stomach and growth model**

The ingested biomass for each time-step (*dt*) was estimated from the encounter rate of prey items multiplied by the probability of capture, the probability of successful approach, and the weight of the prey [2]. The total ingested biomass was stored in the larval gut/stomach [2] and could not exceed the stomach capacity (6% of dry-weight; E. Broughton, NMFS, Woods Hole, USA, personal communication). We also estimated the time spent on feeding using Holling’s disc equation [20]. The routine metabolism $R\text{=}57.12\times10^{\text{-}7}\cdot w^{0.9}\cdot\exp\text{(}0.088\cdot T\text{)}$ (*R*, mg⋅day^-1^) of larval cod was parameterized as a function of larval dry mass (*w*, mg) and temperature (*T*) [21]. The metabolism increased during larval activity (e.g. foraging) and we defined active metabolism as *R_a_*_ctive_=2.5*R* for larval size *SL*≥5.5 mm and *R_a_*_ctive_=1.4*R* for larval size *SL*<5.5 mm [14]. Foraging and a general activity level occurred while the light level exceeded the threshold of 0.01 µmol s^-1^ m^-2^. Below the light threshold, active metabolism equaled routine metabolism (*R_a_*_ctive_=*R*). For each time step, growth was calculated as either temperature and food dependent or only temperature dependent. Temperature-dependent growth took place if the amount of biomass stored in the stomach exceeded or equaled the biomass required for maximum growth. The temperature-dependent growth rate was [22]:

$SGR(w,T)=a-b\ln(w)-c\ln(w)^{2}+d\ln(w)^{3}$ 2

Here, *SGR* is specific growth rate (% day^-1^) and is a function of temperature (*T*) and dry weight (*w*, mg), and where $\text{a}=1.08+\text{1.79T, b}=\text{0.074T, c}=\text{0.0965T, d}=\text{0.0112T}$. The instantaneous growth rate *g* (day^-1^) is then $g=\ln((SGR\cdot dt)/100+1)/dt$ [23]. Total biomass (dry-weight) necessary to grow at maximum temperature-dependent rate is then:

$D=(\exp(g\cdot dt)-1)\cdot w_{t-1}+R_{active}\cdot dt)/A$ 3

Here, *A* (dimensionless) is the assimilation efficiency [24], and *w_t-1_* is the larval weight calculated at the previous time step. If the stomach content (*s_t_* in mg) is lower than required (*D*) by the temperature-dependent growth (*SGR*), growth is considered food-limited and therefore constrained by the biomass available in the stomach. Available stomach volume at the current time step (*s_t_*) is a function of the ingested material (*i*), the remaining stomach content from last time step (*s_t-1_*), and the biomass extracted for growth (*D*):

$s_{t}=s_{t-1}-D+i$ 4

And finally, the larval weight (*w_t_*) at the current time step (*t*) is given by

$w_{t}=\left\{\begin{matrix} w_{t-1}\cdot\exp(g\cdot dt) if D\leq s_{t} \\ s_{t}A-R _{active}\cdot dt if D>s_{t} \end{matrix}\right.$ 5

**Mortality model**

Mortality rate in the water column at a given depth and time of the year, and day was a combination of mortality from piscivores (visual predators), invertebrates (size dependent predators), and from starvation. The piscivore predation (*m_f_*, h^-1^) can be considered a generic mortality term from a fixed number (1 fish in 100 m^3^) of visual predators [25]. The visual predation was assumed to be proportional to the visual range squared *m_f_*= 0.05·*P^2^* [25], where *P* (mm) was the light- and prey size-dependent perception distance of the piscivores (the coefficient 0.05 includes all factors such as fish density and escape probability [25]). The mortality rate from piscivores changed with light intensity as their eyes depend on light, and therefore varied through the day and with depth. The invertebrate predation (*m_n_*, h^-1^), *m_n_*= 0.01·*SL^-1.3^*, was constant with depth and time of the day and decreased with larval size [26]. *SL* denotes the larval size in mm. The starvation mortality (*m_s_*, h^-1^) was triggered when the cost of energy to sustain metabolism exceeded the biomass available in the stomach and was set to a fixed rate *m_s_*,=1e-6. If the weight of an individual larva was lower than 70% of its expected weight at length [22], the larva was considered to have died and the probability of survival was set to zero. Total instantaneous mortality (*m_z_* ,h^-1^) rate was defined as *m_z_* = *m_n_* + *m_f +_* *m_s_*

**Larval vertical behavior model**

Diel vertical migration resulted from assuming that cognitive larval decision making can take place in the water column. We allowed the larva to “sense” the potential ingestion and mortality rates above and below the larval current depth position [3,27,28]. For each time-step, the larval swimming speed [29] times the time-step length defined the maximum distance vertically (up and down) from its current depth position the larva could potentially move to. The size dependent function for routine swimming speed *v* (mm/s) was [29]:

$v(SL)=0.261\cdot(SL^{(1.552\cdot SL^{(0.920-1.0)})})-(5.289/SL)$ 6

If the larva did swim to a new depth position, an extra energy cost was added to the bio-energetic calculations. This cost was 20% of routine metabolism if the larva swam the maximum distance allowed otherwise it was scaled according to distance. The larva selected a new depth position ($z_{i}^{*}$) where the ingestion rate was maximized and mortality rate was minimized - a trade-off between ingestion and predation rate. A hungry larva would take more risk to find food compared to a larva with a full gut. Willingness to take risk (*π_i,t_* ∈ [0-1]) was estimated as a function of stomach fullness and larval size. For each available new depth position above and below the current depth position of the larva we estimated the possible local mortality (*m*) and ingestion rates (*F_z_,* h^-1^). The selected depth, $z_{i}^{*}$, where the combination of willingness to take risk, and maximizing ingestion rate, while minimizing predation for larval individual *i* results from maximizing

$z_{i}^{*}(t)=\max_{z}[(1-\pi_{i,t})F_{z}-\pi_{i,t}m_{i}]$ 7

Here

$\pi_{i,t}=\left\{\frac{\left(\frac{s_{i,t}-STV}{1-STV}\right)^{^{\beta}},s_{i,t}>STV}{0 ,s_{i,t}\leq STV}\right\}$ 8

*β*=5, *s* is stomach fullness which ranges from 0 (empty) to 1 (full), $STV(SL)=0.3+10^{3}\cdot(1+SL\cdot e^{SL})^{-1}$ is the size-dependent (*SL* (mm) is standard length) stomach threshold value (*STV*), which defined the normalized hunger level. *STV* ranges from 0.7 for larval cod of 6 mm length to 0.3 for larval cod of 18 mm length [2,27].

**References**

1. Fiksen Ø, MacKenzie BR (2002) Process-based models of feeding and prey selection in larval fish. Marine Ecology Progress Series 243: 151-164.

2. Kristiansen T, Fiksen Ø, Folkvord A (2007) Modelling feeding, growth, and habitat selection in larval Atlantic cod (*Gadus morhua*): observations and model predictions in a macrocosm environment. Canadian Journal of Fisheries and Aquatic Sciences 64: 136-151.

3. Kristiansen T, Lough RG, Werner FE, Broughton EA, Buckley LJ (2009) Individual-based modeling of feeding ecology and prey selection of larval cod on Georges Bank. Marine Ecology Progress Series 376: 227-243.

4. Carton JA, Giese BS, Grodsky SA (2005) Sea level rise and the warming of the oceans in the SODA ocean reanalysis Journal of Geophysical Research 110, C09006.

5. Levitus S, Boyer TP (1994) World Ocean Atlas 1994, Salinity. NOAA Atlas NESDIS 4. U.S. Department of Commerce, NOAA, NESDIS.

6. O'Brien WJ, Evans BI, Browman HI (1989) Flexible search tactics and efficient foraging in saltatory searching animals. Oecologia 80: 100-110.

7. Hunter JR (1981) Feeding ecology and predation of marine fish larvae. In: Lasker R, editor. Marine Fish Larvae: Morphology, Ecology, and Relation to Fisheries. Seattle: University of Washington Press. pp. 33-77.

8. Aksnes DL, Utne ACW (1997) A revised model of visual range in fish. Sarsia 82: 137-147.

9. Skartveit A, Olseth JA, Tuft ME (1998) An hourly diffuse fraction model with correction for variability and surface albedo. Solar Energy 63: 173-183.

10. Riley GA (1956) Oceanography of the Long Island Sound 1952–1954. II Physical oceanography Bull Bing Oceanogr Coll 15: 15-46.

11. Mueter FJ, Broms C, Drinkwater KF, Friedland KD, Hare JA, et al. (2010) Ecosystem responses to recent oceanographic variability in high-latitude Northern Hemisphere ecosystems. Progress in Oceanography 81: 93-110.

12. Orlova EL, Boitsov VD, Dolgov AV, Rudneva GB, Nesterova VN (2005) The relationship between plankton, capelin, and cod under different temperature conditions. ICES Journal of Marine Science 62: 1281-1292.

13. Hátún H, Payne MR, Beaugrand G, Reid PC, Sandø AB, et al. (2009) Large bio-geographical shifts in the north-eastern Atlantic Ocean: From the subpolar gyre, via plankton, to blue whiting and pilot whales. Progress in Oceanography 80: 149-162.

14. Lough RG, Buckley LJ, Werner FE, Quinlan JA, Edwards KP (2005) A general biophysical model of larval cod (*Gadus morhua*) growth applied to populations on Georges Bank. Fisheries Oceanography 14: 241-262.

15. Daewel U, Peck MA, Schrum C, St John MA (2008) How best to include the effects of climate-driven forcing on prey fields in larval fish individual-based models. J Plankton Res 30: 1-5.

16. MacKenzie BR, Kiørboe T (2000) Larval fish feeding and turbulence: A case for the downside. Limnology and Oceanography 45: 1-10.

17. MacKenzie BR, Kiørboe T (1995) Encounter rates and swimming behavior of pause-travel and cruise larval fish predators in calm and turbulent laboratory environments. Limnology and Oceanography 40: 1278-1289.

18. Kiørboe T, MacKenzie B (1995) Turbulence-enhanced prey encounter rates in larval fish: Effects of spatial scale, larval behaviour and size. Journal of Plankton Research 17: 2319-2331.

19. Caparroy P, Thygesen UH, Visser AW (2000) Modelling the attack success of planktonic predators: patterns and mechanisms of prey size selectivity. Journal of Plankton Research 22: 1871-1900.

20. Holling CS (1966) The functional response of invertebrate predators to prey density. Mem Entomol Soc Can 48: 1-86.

21. Finn N, Rønnestad I, van der Meeren T, Fyhn HJ (2002) Fuel and metabolic scaling during the early life stages of Atlantic cod *Gadus morhua*. Marine Ecology Progress Series 243: 217-234.

22. Folkvord A (2005) Comparison of size-at-age of larval Atlantic cod (*Gadus morhua*) from different populations based on size- and temperature-dependent growth models. Canadian Journal of Fisheries and Aquatic Sciences 62: 1037-1052.

23. Otterlei E, Nyhammer G, Folkvord A, Stefansson SO (1999) Temperature- and size-dependent growth of larval and early juvenile Atlantic cod (*Gadus morhua*): a comparative study of Norwegian coastal cod and northeast Arctic cod. Canadian Journal of Fisheries and Aquatic Sciences 56: 2099-2111.

24. Buckley LJ, Dillmann DW (1982) Nitrogen utilization by larval summer flounder, *Paralichthys dentatus* (Linnaeus). Journal of Experimental Marine Biology and Ecology 59: 243-256.

25. Fiksen Ø, Aksnes DL, Flyum MH, Giske J (2002) The influence of turbidity on growth and survival of fish larvae: a numerical analysis. Hydrobiologia 484: 49-59.

26. McGurk MD (1986) Natural mortality of marine pelagic fish eggs and larvae - Role of spatial patchiness. Marine Ecology-Progress Series 34: 227-242.

27. Kristiansen T, Jørgensen C, Lough RG, Vikebø F, Fiksen Ø (2009) Modeling rule-based behavior: habitat selection and the growth-survival trade-off in larval cod. Behavioral Ecology 20: 490-500.

28. Vikebø F, Jørgensen C, Kristiansen T, Fiksen Ø (2007) Drift, growth and survival of larval Northeast Arctic cod with simple rules of behaviour. Marine Ecology Progress Series 347: 207-219.

29. Peck MA, Buckley LJ, Bengtson DA (2006) Effects of temperature and body size on the swimming speed of larval and juvenile Atlantic cod (*Gadus morhua*): Implications for individual-based modelling. Environmental Biology of Fishes 75: 419-429.
